# Plant Adaptogens—History and Future Perspectives

**DOI:** 10.3390/nu13082861

**Published:** 2021-08-20

**Authors:** Velislava Todorova, Kalin Ivanov, Cédric Delattre, Vanya Nalbantova, Diana Karcheva-Bahchevanska, Stanislava Ivanova

**Affiliations:** 1 Department of Pharmacognosy and Pharmaceutical Chemistry, Faculty of Pharmacy, Medical University-Plovdiv, 4002 Plovdiv, Bulgaria; kalin.ivanov@mu-plovdiv.bg (K.I.); vanya.nalbantova@mu-plovdiv.bg (V.N.); diana.karcheva@mu-plovdiv.bg (D.K.-B.); stanislava.ivanova@mu-plovdiv.bg (S.I.); 2 Université Clermont Auvergne, Clermont Auvergne INP, CNRS, Institut Pascal, 63000 Clermont-Ferrand, France; cedric.delattre@uca.fr; 3 Institut Universitaire de France (IUF), 1 rue Descartes, 75005 Paris, France

**Keywords:** plant adaptogens, *Panax ginseng*, *Eleuterococcus senticosus*, *Rhaponticum carthamoides*, *Rhodiola rosea*, *Schisandra chinensis*

## Abstract

Adaptogens are synthetic compounds (bromantane, levamisole, aphobazole, bemethyl, etc.) or plant extracts that have the ability to enhance the body’s stability against physical loads without increasing oxygen consumption. Extracts from *Panax ginseng*, *Eleutherococcus senticosus*, *Rhaponticum carthamoides*, *Rhodiola rosea*, and *Schisandra chinensis* are considered to be naturally occurring adaptogens and, in particular, plant adaptogens. The aim of this study is to evaluate the use of plant adaptogens in the past and now, as well as to outline the prospects of their future applications. The use of natural adaptogens by humans has a rich history—they are used in recovery from illness, physical weakness, memory impairment, and other conditions. About 50 years ago, plant adaptogens were first used in professional sports due to their high potential to increase the body’s resistance to stress and to improve physical endurance. Although now many people take plant adaptogens, the clinical trials on human are limited. The data from the meta-analysis showed that plant adaptogens could provide a number of benefits in the treatment of chronic fatigue, cognitive impairment, and immune protection. In the future, there is great potential to register medicinal products that contain plant adaptogens for therapeutic purposes.

## 1. Introduction

Adaptogens are pharmacologically active compounds or plant extracts from different plant classes (for example: Araliaceae—*Panax ginseng*, *Eleutherococcus senticosus*, Asteraceae—*Rhaponticum carthamoides*, Crassulaceae—*Rhodiola rosea*, and Schisandraceae—*Schisandra chinensis*) [1,2,3]. They have the ability to enhance the body’s stability against physical loads without increasing oxygen consumption. The intake of adaptogens is associated not only with the body’s better ability to adapt to stress and maintain/normalize metabolic functions, but also with better mental and physical performance [1,2,3].

There are two main classes of adaptogens. The first class includes plant adaptogens, while the other includes synthetic adaptogens, which are also called actoprotectors.

Although plant adaptogens have been used by people since ancient times, the term “adaptogen” is relatively young—it was introduced in 1947 by the Soviet scientist Lazarev [1,4]. It defines adaptogens as substances that cause non-specific resistance of the living organisms [1,2,4]. Adaptogens have a positive effect on humans and animals. The use of plant adaptogens has a rich history. They have been used by man for hundreds of years in different parts of the world, while data on the use of the first synthetic adaptogen, bemethyl, date back to the 1970s. Bemethyl was introduced in the 1970s by Professor Vladimir Vinogradov. Since then, numerous synthetic adaptogens have been developed: bromantane, levamisole, aphobazole, chlodantane, trekrezan. Their intake is associated not only with increased physical and mental resistance, but also with vasodilation and decreased blood sugar and lactate [3]. They are widely used in sports medicine, but since 2009, WADA has included bromantane in the prohibited list and since 2018, bemethyl has also been included in the monitoring program of WADA [5,6].

In 1980, the scientists Breckham and Dardimov found that adaptogens increase the body’s resistance not only to physical but also to chemically and biologically harmful agents [1,2,7], which further expands the potential of their use.

Breckham and Dardimov systematized the plants with adaptogenic properties. These are: *Panax ginseng*, *Eleutherococcus senticosus*, *Rhaponticum carthamoides*, *Rhodiola rosea*, and *Schisandra chinensis* [7].

Although plant adaptogens have been used for centuries, their effects continue to be studied to this day. They also have promising potential for wider applications in the future.

The biological effects of plant adaptogens are related to the complex of biologically active compounds they contain. Plant adaptogens have very rich phytochemical composition. Some of the most important phytochemicals with adaptogenic properties are: triterpenoid saponins (in *Panax ginseng*—ginsenosides; in *Eleutherococcus senticosus*—eleutherosides); phytosterols and ecdysone (in *Rhaponticum carthamoides*); lignans (in *Schisandra chinensis*); alkaloids; flavonoids, vitamins, etc. [4,8,9].

The mechanism of action of the plant adaptogens is complex and is not fully understood. Recent studies report that the intake of plant adaptogens like extracts of *Eleutherococcus senticosus* root, *Schisandra chinensis* root, *Rhodiola rosea* root is associated with affecting the hypothalamic—pituitary—adrenal axis [10] and some stress mediators [11]. Moreover, the intake of such extracts affects nitric oxide levels, lactate levels, blood glucose levels, cortisol levels, plasma lipid profile, hepatic enzymes, etc. [8,11,12,13,14].

Current and potential uses of these medicinal plants are associated with mental diseases and behavioural disorders, cognitive function and stress-induced diseases (anxiety, cardiovascular diseases, diabetes) [4,9,15,16,17,18,19,20]. The intake of plant adaptogens is not associated with serious side effects [20,21].

The aim of our study is to evaluate the data about the use of adaptogens in the past and the future perspectives of their use.

## 2. Materials and Methods

The first step of the screening process included identifying eligible studies. An expanded search for articles without language restrictions was conducted on the following databases: Google Scholar, PubMed, and Web of Science. In the search process, the following search key words were used: “adaptogens”, “actoprotectors”, “plant adaptogens”, “*Panax ginseng*”, “*Eleutherococcus senticosus*”, “*Schisandra chinensis*”, “*Rhodiola rosea*”, “*Rhaponticum carthamoides*”, “*Leuzea carthamoides*”, “ginseng adaptogen properties study”, “*Eleutherococcus senticosus* adaptogen properties study”, “randomized controlled study *Eleutherococcus senticosus*”, “*Leuzea carthamoides* adaptogen properties study”, “*Rhaponticum carthamoides* adaptogen properties study”, "ecdysterone adaptogen properties study”, “20-hydroxyecdisone adaptogen study”, “ecdysterone from Leuzea adaptogen studies”, “*Leuzea carthamoides* adaptogen properties in rats”, “performance gain *Rhaponticum carthamoides*”, “*Rhodiola rosea* in sport”, “*Rhodiola rosea* adaptogen properties study”, “*Schisandra chinensis* in sport”, “*Schisandra chinensis* randomized study”, “*Schisandra chinensis* adaptogen properties study”, and “*Schisandra chinensis* mice study”.

Further records concerned the history of plant adaptogens, botanical and phytochemical characteristics, and application of adaptogens. 

**Figure 1:**
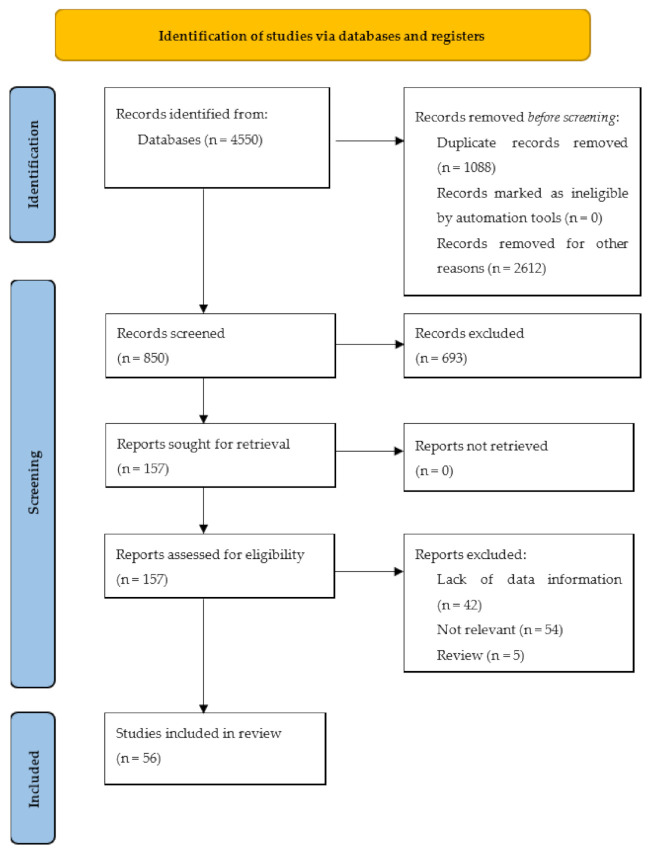
PRISMA 2020 flow diagram for new systematic reviews which included searches of databases and registers only.

In the second step, we followed the PRISMA (Preferred Reporting Items for Systematic Reviews and Meta-Analysis) presented in Figure 1 [22], guide for a systematic review.

In the third step, the relevant studies were chosen based on exclusion and inclusion criteria. The exclusion criteria were: (i) articles written in a language other than English or Russian; (ii) webinars or blogs; (iii) articles with irrelevant topics, and (iv) lack of data information. Inclusion criteria were: (i) human studies; (ii) animal studies, and (iii) studies investigating the impacts of adaptogens.

During the fourth step, the selected full articles were read and identified.

In total, 56 studies were selected and included in the present review.

## 3. Results and Discussion

### 3.1. Panax Ginseng

**Figure 2:**
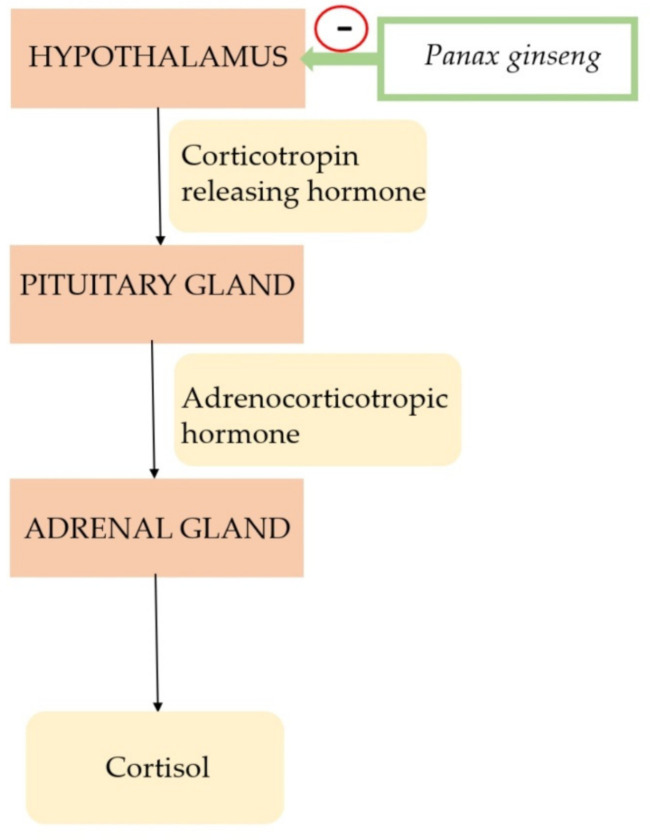
Mechanism of action of *Panax ginseng*.

The first evidence of the use of *Panax ginseng* (*Panax ginseng* C.A. Mey.) dates back more than 2000 years [23]. In the past, the stems, leaves, and mainly the roots of ginseng were used. Extracts were prepared and used to maintain homeostasis in the human body, treat fatigue and weakness, increase immune protection, and treat hypertension, diabetes type 2, and erectile dysfunction [2,23,24,25,26]. In Chinese traditional medicine, ginseng extracts have also been used as nootropic agents and as a tonic [24,25,26]. The exact mechanism of adaptogenic action of *Panax ginseng* is unknown, but it is supposed that it affects the hypothalamic-pituitary-adrenal axis in Figure 2 and the antioxidant effect [24,27,28,29].

*Panax ginseng* is an example of a medicinal plant, widely used in ancient times, but also with great application today. It is also one of the plants defined as a natural adaptogen.

*Panax ginseng* has a rich botanical phytochemical composition. Today, more than 200 substances isolated from Korean ginseng are known, ginsenosides predominantly: Ra1, Ra2, Ra3, malonyl-G-Rb1, malonyl-G-Rb2, malonyl-G-Rc, malonyl-GRd, Rs1, Rs2, Rs3, Rg3, Rg5, Rh2, K-R2, Rf, Rf2, 20(R)-G-Rg2, Rg6, 20(R)-G-Rh1, 20(E)-G-F4, Rh4, K-R1, and poly-acetyleneginsenoside-Ro [30].

It is considered that the adaptogenic properties of *Panax ginseng* extract are due to the ginsenosides [27].

Nowadays, numerous products containing ginseng extracts are available. Most of these products are sold as food supplements but there are also many over-the-counter medicines. Ginseng radix is included in European Pharmacopeia and in the US Pharmacopeia. The principal root has a cylindrical form, sometimes branched, up to about 20 cm long and 2.5 cm in diameter. Its surface is pale yellow/cream in white ginseng, while it is brownish-red in red ginseng. The rootlets, which are many in the lower part of white ginseng, are usually absent in red ginseng. Reduced to a powder, it is light yellow. Ginseng dry extract is produced from the root by a suitable procedure using a hydroalcoholic solvent equivalent in strength to ethanol. The main compounds that can be detected in the extract are: ginsenoside Rg1, ginsenoside Re, ginsenoside Rf, ginsenoside Rb1, ginsenoside Rc, ginsenoside Rd, ginsenoside Rb2 [31].

Although the extract has been used for more than two millenniums, there are a limited number of clinical studies that have investigated the benefits or the side effects of its use. More double-blind randomized studies are needed to be performed.

In Table 1, we have summarized the main benefits of the *Panax ginseng* extract intake.

The data suggest that an intake of *Panax ginseng* extracts is associated with ergogenic effects and enhanced muscle strength. The inclusion of *Panax ginseng* in the diet of athletes would help increase the body’s physical resilience and help the body recover between workouts.

Intake of *Panax ginseng* extract is associated also with improved plasma lipid profile and blood glucose level. Extracts of *Panax ginseng* might be included in the diet of patients with cardiovascular diseases, hyperlipidemia, and diabetes. Extracts from the plant not only increase cognitive function and memory functions, but also improve sleep and fatigue.

We did not find multicenter randomized double-blind studies including the intake of *Panax ginseng*. For better exploration of the benefits and future applications of the *Panax ginseng* extract, multicenter randomized double-blind studies should be performed. The plant has a great potential to be included in medicines for treatment of different conditions. 

The intake of *Panax ginseng* extract is not associated with serious side effects [21,32,33,34,35,36,37,38,39,40,41]. The safety of the extract is another benefit to be considered.

### 3.2. Eleutherococcus Senticosus

Siberian ginseng (*Eleutherococcus senticosus* ((Rupr. and Maxim.) Maxim.) was first described by Porfiry Kirilov in the 19th century [42]. Its adaptogenic effects were widely studied in Russia between 1960 and 1970 [43]. The first data on the use of plant extracts in athletes were reported from Russia. Today, plant extracts are used not only by athletes around the world, but also by many other consumers who are not actively involved in sports.

The phytochemical composition consists of phenylpropanoid—syringin; lignans—sesamin; saponins—daucosterol; coumarins, terpenoids, flavonoids, organic acids, and vitamins [44,45]. Extracts of *Eleutherococcus senticosus* root are obtained, stimulating the immune system, influencing adaptation against external factors, improving mental and physical conditions and memory functions, and have a hypoglycaemic effect and are anti-inflammatory [13,45,46]. *Eleutherococcus senticosus* is thought to exert its adaptogenic effects by influencing the hypothalamic-pituitary-adrenal axis [44].

Most of the extracts of *Eleutherococcus senticosus* are prepared by the roots. The rhizome of *Eleutherococcus senticosus* is described in European pharmacopeia. The rhizome has a diameter of 1.5 to 4 cm and an irregular cylindrical shape. The surface is longitudinally wrinkled with grayish-brown to blackish-brown colour [47].

Although *Eleutherococcus senticosus* were described a few hundred years ago, research into its use continues. In Table 2, we summarized results of studies with *Eleutherococcus senticosus*.

The data suggest that the intake of *Eleutherococcus senticosus* supports the physical activity, weight reduction, mental health, and fatigue. Extracts of the plant can be used only for fatigue, but also in the case of disturbed sleep.

The intake of *Eleutherococcus senticosus* extract may help to increase the cognitive function. Siberian ginseng extract could be included in the diet of patients with hyperlipidemia because it has a beneficial effect on the lipid profile. There is a potential for development of medicinal products containing *Eleutherococcus senticosus* extract to be taken by patients with various pathologies such as: obesity, overweight, hyperlipidemia, etc. New nootropic drugs containing a standardized plant extract could also be registered.

We did not find multicenter randomized double-blind studies that included the intake of Siberian ginseng. For better exploration of the benefits and future applications of the *Eleuterococcus senticosus* extract, multicenter randomized double-blind studies should be performed. The plant has a great potential to be included in medicines for treatment of different conditions; moreover, the intake of *Eleuterococcus senticosus* extract is not associated with serious side effects [46,48,49,50,51,52,53,54,55].

### 3.3. Rhaponticum Carthmoides

*Rhaponticum carthamoides* (*Rhaponticum carthamoides* IIjin.) is a perennial herb which has been used for centuries in Russia, China, and Mongolia [14]. The plant is also known as Leuzea. Extracts of the plant have been used to treat weakness [14,56], lung diseases, kidney diseases, fever, and angina [57].

In 1969, Brekhman and Dardymov classified this plant as an adaptogen [14]. The use of products containing *Rhaponticum carthamoides* root extract has increased in recent decades. The extract has many beneficial effects on humans like: enhanced physical endurance and performance, anabolic effect, hypocholesterolemic effects, neuroprotective effect, antidiabetic properties, anti-oxidative, and increased immunity [14,57]. The mechanism by which ecdysteroids act is binding with signal transduction pathways, rather than with steroid and estrogen receptors [58].

In the 1970s, the extracts from Leuzea showed beneficial effects in athletes and their use became a routine practice in the training of many athletes. The intake of Leuzea extract increases the body’s adaptation to various factors, which can be defined as stress for the body [14,57] and at the same time has a good level of safety.

**Figure 3:**
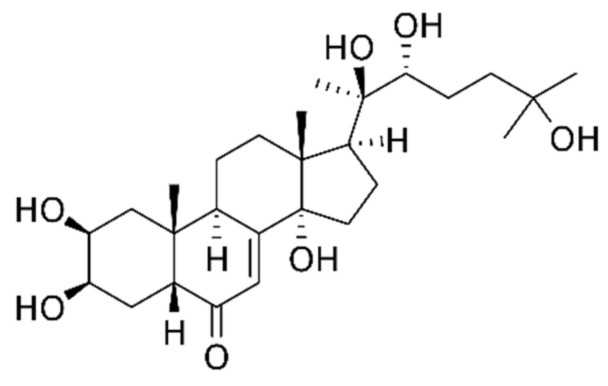
20-hydroxyecdysone structure.

The phytochemical composition of *Rhaponticum carthamoides* is rich of ecdysteroids and phenols. The main ecdysteroid is 20-hydroxyecdysone in Figure 3 [14]. The adaptogenic properties of the extract are related mainly to its presence [14]. Other components of the phytochemical composition are phenols and essential oil [14].

20-hydroxyecdysone has a typical steroidal structure. A recent study, funded by WADA and conducted in 2019, reported 20-hydroxyecdisone as a non-conventional anabolic agent, which can significantly increase the muscle mass. A significant dose-responsive anabolic effect of 20-hydroxyecdysone was reported [59].

In 2020, 20-hydroxyecdysone was included in the WADA monitoring program and it is very likely that the substance will be included in the prohibited list in the next few years [60]. In the past few years, other adaptogens were included in WADA’s prohibited list and monitoring program, because these compounds were considered to improve the performance of the athletes. Bromantane is included in the prohibited list as a non-specified stimulant. Since 2018, bemethyl is monitored by WADA, but it is still not included on the prohibited list [5,6]. The main difference between 20-hydroxyecdysone and these two compounds is that 20-hydroxyecdysone is a natural compound while the other two are synthetic adaptogens. The main reason 20-hydroxyecdysone is monitored by WADA is that the intake of this compound improves the performance of athletes. Nowadays, food supplements containing Leuzea (which contain 20-hydroxyecdysone) are often included in the supplementation of professional athletes.

Table 3 presents studies related to the use and effects of *Rhaponticum carthamoides*.

We expanded our search with animal studies; found results are presented in Table 4.

The data suggest that intake of *Rhaponticum carthamoides* extracts is associated with anabolic effects—increased body mass weight and enhanced muscle strength. Other important benefits are improved mental endurance and improved plasma lipid profiles. Improvement of cardiac and cognitive functions has also been reported.

Because of improvement in cardiac functions, the *Rhaponticum carthamoides* extract might be especially useful for patients with cardiovascular diseases.

Despite the benefits of the extract use, the studies on humans are limited in number and not sufficient for a more complete and comprehensive assessment.

The anabolic effects were also reported in animal studies. The data from animal studies suggests that application of *Rhaponticum carthamoides* is also associated with a neuroprotective effect. 

The intake of *Rhaponticum carthamoides* is not associated with serious side effects [19,59,60,61,62,63,64,65,66,67,68,69,70,71,72].

The data obtained from human and animal studies about synergetic anabolic effect of *Rhaponticum carthamoides* and *Rhodiola rosea* are contradictory. Further studies are needed to confirm the synergetic effect of these adaptogenic plants. 

For better exploration of the benefits and future applications of the *Rhaponticum carthamoides* extract, more in vivo studies and multicenter randomized double-blind studies should be performed.

### 3.4. Rhodiola rosea 

In traditional medicine, its (*Rhodiola rosea* L.) applications are described as an adaptive agent that increases physical endurance, affects fatigue, depression, and disorders of the nervous system. It has been used in the past in Asia to treat flu and colds, and there is reported use in tuberculosis. In the Scandinavian part of Europe, plant extracts have been used to increase physical endurance [73].

Six groups of compounds predominate in the phytochemical composition of the plant: phenylpropanoids, phenylethanol derivatives, flavonoids, phenolic acids, and mono- and triterpene [74].

The main phenylethanol derivatives are salidroside (rhodioloside), para-tyrosol, and phenylpropanoid-rosavin. These are also responsible for the adaptogenic and ergogenic effects of *Rhodiola rosea* [8,73,75].

The adaptogenic effect of *Rhodiola rosea* is associated with activation of the cerebral cortex by increasing norepinephrine and serotonin levels. In addition, it affects the hypothalamic-pituitary-adrenal axis, reducing the levels of corticotropin-releasing hormones, corticotropin, cortisol, and epinephrine [29,73,76].

Studies of *Rhodiola rosea* began with Dioscorides and continues nowadays. In Table 5, we summarized beneficial effects of *Rhodiola rosea* based on studies. 

The data suggest that the intake of the extract of *Rhodiola rosea* is associated with antioxidant and adaptogen properties. *Rhodiola rosea* extract might be used not only for overcoming the fatigue but might be included in the diet of people with heart diseases, because of the beneficial effect on heart rate and muscle contractions. The use of plant extracts is recommended for sleep disorders and anxiety. The hepatoprotective effect of the plant determines the use of its extract in liver diseases. Improving physical strength during exercise and recovery after training are the reasons why *Rhodiola rosea* extracts are also taken by athletes as a supplement to their diet.

The intake of *Rhodiola rosea* is not associated with serious side effects [77,78,79,80,81,82,83,84,85,86].

For better exploration of the benefits and future applications of the *Rhodiola rosea* extract, more multicenter randomized double-blind studies should be performed. The plant has a great potential to be included in medicines for treatment of different conditions.

### 3.5. Schisandra chinensis 

*Schisandra chinensis* (*Schisandra chinensis* (*Turcz.*) Bail) is first described in the book *Shen Nong Ben Cao Jing*, around 200 AD, as a remedy for cough and asthma [87]. In the past, *Schisandra chinensis* fruits and seeds were used to improve night vision, reduce hunger, thirst, and exhaustion. In 1960 in Russia, the adaptogenic properties of the plant were proven [88]. The fruits of *Schisandra chinensis* are used today [87,88,89,90].

According European Pharmacopoeia, Schisandra berry is more or less spherical, up to 8 mm in diameter. It could be red/reddish-brown/blackish, it could be covered in a whitish frost. It has a strongly shriveled pericarp. The seeds are only 1 or 2, yellowish-brown and lustrous. The seed-coat is thin [91]. 

For production of the extract, the fruit should be reduced to a powder. The colour of powder should be reddish-brown.

The Schisandra fruit has a complex phytochemical composition in which the lignans are the major characteristic constituents. Five classes of different lignans are found in the fruits of Schisandra: dibenzocyclooctadiene lignans (type A), spirobenzofuranoid dibenzocyclooctadiene lignans (type B), 4-aryltetralin lignans (type C), 2,3-dimethyl-1,4-diarylbutane lignans (type D), and 2,5-diaryltetrahydrofuran lignans (type E) [88].

The adaptogenic properties of *Schisandra chinensis* are due to the lignin complex, mainly dibenzocycloocstadiene lignans, the main schisandrin [92,93]. Schizandrin was isolated and identified for the first time by N.K. Kochetkov in 1961 [88,92,93].

**Figure 4:**
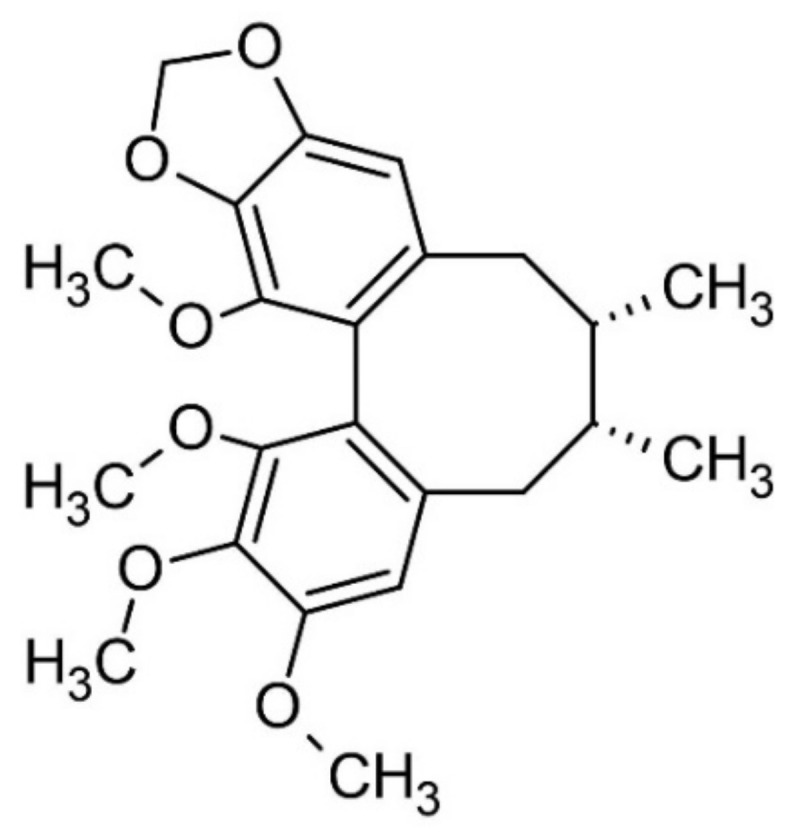
Structure of γ-Schisandrin.

One of methods for identification of the plant, described in the European Pharmacopeia, involves a TLC technique with identification of γ-Schisandrin in Figure 4.

Several studies reported that some lignans (gomisin A, gomisin G, schizandrin, and schisanhenol) possess antitumor bioactivities [94,95,96,97,98].

The Schisandra fruit, particularly the seeds, contains many volatile compounds as well: α-ylangene, α-cedrene, β-chamigrene, and β-himachalene [99,100].

From *Schisandra chinensis* fruit are also isolated polysaccharides, glycosides (dihydrophaseic acid-3-O--d-glucopyranoside, benzyl alcohol-O--d-glucopyranosyl (1→6)--d-glucopyranoside and benzyl alcohol-O--d-glucopyranosyl (1→2)--d-glucopyranoside), organic acids (vitamin C, malic acid, citric acid, and tartaric acid), and vitamin E. [12,87,101,102]. In small quantities, the fruit of *Schisandra chinensis* contained flavonoids such as rutine [103]. Preschsanartanin, schintrilactones A–B, schindilactones A–C, and wuweizidilactones A–F are the new isolated triterpenoids from *Schisandra chinensis* fruit [88].

All of phytochemicals determined beneficial effects such as: cytotoxic, antioxidant, neuroprotective, hepatoprotective, increased physical strength, stress-protective, anti-inflammatory [12,88,104]. The adaptogenic effect of *Schisandra chinensis* is associated with the antioxidant effect and the influence on the hypothalamic-pituitary-adrenal axis by lowering the level of corticotropin-releasing hormone [29,76]. 

Research on the effects of *Schisandra chinensis* continues. 

In Table 6, we present summarized data about beneficial effects of *Schisandra chinensis*.

Discovered trials are insufficient to summarize the use of *Schisandra chinensis*. We expanded our search with animal studies, the results found are presented in Table 7.

The data from animal and human studies suggest that the application of extract of *Schisandra chinensis* is associated with adaptogenic, antioxidant properties, tonic, and stress-protective effect. 

The intake of plant extract can improve memory and concentration. The plant extract has serious potential to be included in medicinal products for use in the treatment of patients with hypercholesterolemia or patients with cardiovascular disease. Of course, the application of the extract would have a number of benefits in healthy patients due to its pronounced antioxidant properties.

The positive effect of the plant on blood sugar and liver enzymes suggests the inclusion of *Rhodiola rosea* extracts in the treatment of patients with diabetes and liver disease. In recent decades, *Schisandra chinensis* extract has been taken by athletes to increase physical activity and the body’s adaptation to stress.

The studies on humans are limited in number, so a full assessment of the effects is difficult to make.

The intake of *Schisandra chinensis* is not associated with serious side effects [105,106,107,108,109,110,111,112,113,114,115,116].

For a better exploration of the benefits and future applications of the *Schisandra chinensis* extract, more in vivo studies and multicenter randomized double-blind studies should be performed.

## 4. Conclusions

The natural adaptogens have the ability to increase the body’s resistance to stress changes caused by different types of stressors. Unlike the synthetic adaptogens, the natural are extracts with an extremely rich phytochemical composition. Their adaptogenic properties are not due to one molecule, but to the combination of different substances. The use of natural adaptogens by humans has a rich history—they have been used in recovery from illness, physical weakness, impaired mental function, and other conditions. For about 50 years, plant adaptogens have been used by professional athletes due to their high potential to increase the body’s resilience and improve physical endurance. Nowadays, some of the most used plant adaptogens are *Panax ginseng, Eleutherococcus senticosus*, and *Rhaponticum carthamoides*. Since 2020, ecdysterone, which is rich in Leuzea extract, has been included in WADA’s monitoring program, with prospects for inclusion in the prohibited list. Studies examining the benefits of using extracts of *Rhodiola rosea, Eleutherococcus senticosus, Panax ginseng, Schisandra chinensis* and *Rhaponticum carthamoides* are a limited in number. However, there are potentials for the inclusion of the extracts of these plants in medicinal products aimed at treating chronic fatigue, cognitive impairment, as well as boosting immune defenses. Double-blind randomized multicenter studies would be extremely valuable in evaluating the use of the extracts in patients with cardiovascular disease, in patients with compromised immunity, and in patients with chronic fatigue.

## Figures and Tables

**Table 1:** *Panax ginseng* studies.

Study Objectives	Study Design	Main Results	References
Evaluation of the effects on subjective mood and memory of a single and sub-chronic *Panax ginseng* dose.	Thirty adults, aged 22.87 ± 4.01 years, participated in the study. They received a placebo, 200 or 400 mg *Panax ginseng* extract per day for 3 treatments—8 days with 6 days washout. Period of the study—32 days.	Improved calmness, mood, and mental health.	[21]
Examine the effects of *Panax ginseng* on the lipid profile.	Eight men, aged 21.1 ± 2.1 years, participated in the study. All participants received 2 g of ginseng extract 3 times per day. Period of the study—8 weeks.	Decreased levels of serum total cholesterol (TC), triglycerides (TG), low-density lipoprotein (LDL), plasma malondialdehyde (MDA), and an increase in high-density lipoprotein (HDL).	[32]
Examine the effects on endurance performance of acute supplementation of *Panax ginseng*.	Twelve men, aged 20–24 years, participated in the study. All participants received 200 mg ginseng extract or a placebo one hour before the exercise.	Increased endurance time, blood glucose and insulin levels, catalase, superoxide-dismutase, and total thiol.	[33]
Evaluation of benefits on fatigue in multiple sclerosis with *Panax ginseng* treatment.	Fifty-two women, aged 18–50 years, participated in the study. There were 26 participants who received 500 mg daily of Korean ginseng tablets and 26 were in the placebo group.Period of the study—3 months.	Reduced fatigue. Improved quality of life.	[34]
Examine the effects of *Panax ginseng* on work performance.	Nineteen women, 21–35 years of age, participated in the study. There were 10 participants who received 200 mg of *Panax ginseng* extract daily, and nine of them were in the placebo group. Period of the study—2 months.	No change in maximal work performance, oxygen consumption (VO2), respiratory exchange ratio, minute ventilation, heart rate, and blood lactic acid levels.	[35]
Evaluation of the efficacy of a combination of *Panax ginseng* and vitamins on physical and mental stress.	One-hundred and fourteen women and men, aged 30–60 years, participated in the study. There were 59 participants who received 200 mg daily of ginseng dried extract and vitamins; 55 were in the placebo group. Period of the study—8 weeks.	Increased quality of life, without a difference in blood pressure and heart rate.	[36]
Evaluation of the efficacy of *Panax ginseng* extract on physical and mental performance.	Fifteen men, aged 19.07 ± 0.62 years, participated in the study. Seven of them received 200 mg daily of ginseng extract; eight of the participants were in the placebo group. Period of study—6 weeks.	Increased lactate levels. No change in VO2 and heart rate. Decreased cortisone levels and no change in testosterone levels.	[37]
The influence of *Panax ginseng* on cortisol, growth hormone, and lactate.	Ten women, aged 23.4 ± 0.69 years, participated in the study. In the first 4 weeks, five received 100 mg per day and five were in the placebo group. In the second 4 weeks, they were switched.Period of study—8 weeks.	Increased cortisol. No change in growth hormone and lactate.	[38]
Evaluation of the effects of *Panax ginseng* on sleep.	Fifteen men, aged 19–25 years, participated in the study. There were eight participants who received 4.5 g of ginseng extract daily; seven participants were in the placebo group. Period of the study—2 weeks.	Increased deep sleep. Decreased shallow sleep.	[39]
Evaluation of anti-fatigue effects of *Panax ginseng*.	Eighty-eight men and women 20–60 years of age participated in the study. There were 30 participants who received 1 g of ginseng extract daily; 29 participants had an intake of 2 g of ginseng extract daily. There were29 participants who were in the placebo group. Period of study—2 months.	Reduced the severity of fatigue. Increased glutathione reductase and total glutathione.	[40]
Assessment of an ergogenic effect on Malaysian population of *Panax ginseng* in humid and hot conditions.	Nine men, 25.4 ± 6.9 years of age, participated in the study. In the first trial, they had an intake of 200 mg of *Panax ginseng* one hour before an exercise test and in the second trial, they had an intake placebo.	Decreased lactate, plasma glucose, plasma insulin. Increased free fatty acids. No change in heart rate, VO2, skin, and body temperature.	[41]

**Table 2:** *Eleutherococcus senticosus* studies.

Study Objectives	Study Design	Main Results	References
Examine the effects of *Eleutherococcus senticosus* extract on physical working capacity.	Six men, aged 21–22 years, participated in the study. They were in the control group, placebo group, and the group that received 2 mL ethanol extract (125 mg dried extract) *Eleutherococcus senticosus* twice daily. Period of study—8 days.	Increased maximal oxygen uptake, oxygen pulse, total work, and exhaustion time.	[46]
Examine the effects of *Eleutherococcus senticosus* during maximal and submaximal aerobic exercise.	Twenty men and women, aged 37 ± 8 years, participated in the study. There were 10 participants who received 3.4 mL *Eleutherococcus senticosus* extract; 10 participants were in the placebo group. Period of study—8 weeks.	No changes in heart rate, VO2, lactate.	[48]
Assessment of the effects of *Eleutherococcus senticosus* on metabolism, endurance capacity, and cardiovascular functions.	Nine men, aged 19 ± 2.1 years, participated in the study. The participants received 800 mg daily of *Eleutherococcus senticosus* or placebo. Period of study—8 weeks.	Decreased blood glucose levels. Increased VO2, endurance time, heart rate, and free fatty acids.	[49]
Assessment of the impact of *Eleutherococcus senticosus* on quality of life.	Twenty volunteers, aged over 65 years, participated in the study. There were 10 participants who received 300 mg/day Siberian ginseng extract and 10 participants who were in placebo group. Period of study—2 months.	Improved mental health and social functioning, but prolonged use decreased these improvements. Blood pressure was not affected.	[50]
Assessment of the influence of *Eleutherococcus senticosus* on physical fitness and cellular defence.	Forty-six men and women, aged 23–73 years, participated in the study. There were 31 participants who received 75 drops of *Eleutherococcus senticosus* extract daily and 15 participants who received 120 drops of Echinacea extract daily for one month.	Decreased total cholesterol, LDL, triglycerides, free fatty acids, and glucose. Increased maximal oxygen consumption (VO2max).	[51]
Examine the effects of a dietary supplement containing *Eleutherococcus senticosus* extract on burnout symptoms.	Eighty-seven volunteers, aged 27–63 years, participated in the study. There were 44 participants who had an intake of 100 mg dry extract from *Eleutherococcus senticosus*; 43 were in the placebo group. Period of study—12 weeks.	Decreased fatigue score and Beck depression.	[52]
Evaluate the effects of *Eleutherococcus senticosus* extract on stress.	One-hundred and thirty women and men, aged 30–50 years, participated in the study. There were 49 participants who received 120 mg/day of dry *Eleutherococcus senticosus* extract, 40 participants who worked out, and 41 who worked out and took 120 mg/day dry extract. Period of study—2 months.	Improvement in fatigue, exhaustion, sleep, and restlessness.	[52]
Examine physiological reactions from the intake of *Eleutherococcus senticosus* on cyclists.	Nine men, aged 28 ± 2 years, participated in the study. The participants received 1200 mg per day of *Eleutherococcus senticosus* extract for 7 days and the placebo for 7 days.	No difference in respiratory exchange ratio, oxygen consumption, heart rate, perceived exertion, plasma lactate, plasma glucose.	[53]
Evaluate the effects of *Eleutherococcus senticosus* during the training process in fitness.	In the second series of study, 17 men and women participated; 10 of them received *Eleutherococcus senticosus* extract, and 7 participants were in the controlled group.	Decreased body weight. Increased physical endurance and performance.	[54]

**Table 3:** *Rhaponticum carthamoides* studies.

Study Objectives	Study Design	Main Results	References
Evaluation of the effects of an increased dose of *Rhaponticum carthamoides* during the training process.	Twenty women, aged 25–40 years, participated in the study. There were 12 of them whoreceived 5–15 mg/kg/day ecdysterone; 8 were in controlled group.	Decreased body weight. Increased physical endurance and performance. Improvement of cardiac and cognitive function.	[19]
Examine the effect of ecdysterone-containing products on sport physical exercises.	Forty-six men, aged 25.6 ± 3.7 years, participated in the study. There were 12 participants whohad an intake of 200 mg ecdysterone; 10 participants received 800 mg ecdysterone, 12 participants received the placebo, and 12 of the participants were in the control group—they had an intake of 200 mg ecdysterone without training. Period of study—10 weeks.	Ecdysterone increased body weight, muscle mass. Increased power and strength of performance. Without negative effects on creatinine, glutamate–oxaloacetate transaminase, gamma-glutamyl transferase, and glutamate–pyruvate transaminase. Did not affect steroid profile.	[59]
Evaluation of the effectiveness of ecdysterone in athletes.	Twenty-six women aged 18–22 years participated in the study. There were 12 participants who received ecdysterone from 37.5 to 50 mg; 14 participants were in the controlled group. Period of study—9 moths.	Increased VO2 lactate, performance activity.	[61]
Evaluation of the effectiveness of ecdysterone from *Rhaponticum carthamoides* leaves in athletes.	No information—number of participants. The age of the participants ranged between 27–58 years. Participants received 2–3 g *Rhaponticum carthamoides* tea, infusion, tincture, fermented tea without bitterness. Period of study—15 years.	Increased resistance to disease, physical, and mental endurance.	[62]
Assessment of effects of methoxyisoflavone, 20-hydroxyecdysone, and sulfopolysaccharides intake on training adaptation and markers of muscle anabolism and catabolism.	Forty-five men, aged 20.5 ± 3 years, participated in the study. The participants were divided randomly into four groups: the placebo group, the group that received methoxyisoflavone—800 mg daily, the group that received 20-hydroxyecdysone—200 mg/day, and the group that received sulfo-polysaccharides—1000 mg daily. Period of study—8 weeks.	No change in training adaptation and in anabolic and catabolic effect in training.	[63]
Evaluation of the effects of the combination of *Rhaponticum carthamoides* and *Rhodiola rosea* on performance fatigability and reactions before and after training.	Twenty-seven men, aged 22.3 ± 4.1 years, participated in the study. The participants received a 350 mg tablet which contains 70:30 *Rhaponticum carthamoides* extract and *Rhodiola rosea* extract, or a tablet containing 175 mg maltodextrin, and 175 mg *Rhaponticum carthamoides* and *Rhodiola rosea* extract in ratio 70:30 or placebo.	No change in muscle strength and total work.	[64]

**Table 4:** *Rhaponticum carthamoides* animal studies.

Study Objectives	Study Design	Main Results	References
Evaluating *Rhaponticum carthamoides* effects—growth, increased body weight, and behaviour on rats.	For study, 60 rats were used, divided into 10 groups with six rats in group. Females were fed with 5% Raponticum hay meal, males were fed with 10–20% Raponticum hay meal. Period of study—21 days.	Increased growth and body weight.	[65]
Evaluating *Rhaponticum carthamoides* effects on lipid profile on rats with cerebral ischemia.	For the study, 18 rats were used and received 150 mg/kg *Rhaponticum carthamoides* extract or placebo. Period of study—5 days.	Decreased lysophospholipids in erythrocyte membrane. Increased total lipids and phospholipids.	[66]
Examine ecdysterone neuroprotective mechanism of action.	For the study, 35 rats were used, aged 6–8 weeks. They were divided into seven groups: non-operated, two controlled groups, and three experimental groups. In the experimental groups, the rats were administrated ecdysterone in 5 mg/kg, 10 mg/kg, 20 mg/kg. Period of study—7 days.	Removed glutamatergic excitotoxicity. Neuroprotective effect.	[67]
Examine the anti-obesity effect of 20-hydroxyecdysone.	For the study, 60 rats were used, aged 3 months. They were divided into 12 rats in each group and three of the groups were treated with 18, 56, and 116 mg per day 20-hydroxyedysone. Period of study—3 months.	Decreased LDL. Increased muscle mass. No changes in thyroid-stimulating hormone (TSH), tetraiodothyronine (T4), and triiodothyronine (T3).	[68]
Examine anabolic effects of *Leuzea carthamoides* in quails.	For the study, 1000 quails were used. They were divided into six groups—the control group, four groups that had received a standard diet combined with Leuzea seed 0.2—5%, and the other two groups. Period of study—50 days.	Increased body mass and growth.	[69]
Examine anabolic effects of 20-hydroxyecdysone in quails.	For the study, 200 quails were used; 160 were in the control group. There were 10 that received 20 mg/kg, 10 that received 100 mg/kg, and 10 that received 500 mg/kg 20-hydroxyecdysone isolated from *Leuzea carthamoides*. Period of study—4 weeks.	Increased growth. Anabolic effect.	[70]
Examine the improving memory effect of 20-hydroxyecdysone.	For the study, 80 rats were used, divided into two groups: 10 were in the control group, 70 in the experimental group. They received 1, 10, and 100 mg/kg 20-hydroxyecdysone per day. Period of study—12 weeks.	Induced superoxide dismutase (SOD), catalase, glutathione peroxidase (GSH-Px), and glutathione reductase (GR). Decreased glucose levels, nuclear factor-kB (NF-kB).	[71]
Examine the effects of *Rhapoticum carthamoides*, *Rhodiola rosea*, and their combination on resistance exercise and mechanical power.	For the study, 56 rats were used. Rats were divided into seven groups, eight rats in each group: control group, a group that received only *Rhodiola rosea* extract, a group that received only *Rhaponticum carthamoides* extract, and four groups that received a combination of *Rhaponticum carthamoides* and *Rhodiola rosea* extracts in different quantitative ratios.	*Rhaponticum carthamoides* extract increased muscle protein synthesis. The combination of *Rhaponticum carthamoides* and *Rhodiola rosea* increased muscle protein synthesis and mean power performance.	[72]

**Table 5:** *Rhodiola rosea* studies.

Study Objectives	Study Design	Main Results	References
Studying the effects of short-term supplementation with *Rhodiola rosea*.	Eleven women, aged 19.4 ± 0.8 years, participated in the study. They had an intake of 1.5 g/day *Rhodiola rosea* extract or placebo for 3 days. A 500 mg additional dose of *Rhodiola rosea* extract was taken before each trial.	Increased anaerobic capacity, anaerobic power, and total work. No change in fatigue index.	[77]
Examine hormonal and oxidative stress of *Rhodiola rosea* supplementation and the effects on mental and physical performance.	Twenty-six men participated in the study. Thirteen of them had an intake of 600 mg/day extract of *Rhodiola rosea* and 13 were in placebo group. Period of study—4 weeks.	Improved reaction and response time. Increased antioxidant capacity. Without changes in hormone profile and endurance exercise capacity.	[78]
Examine the levels of inflammatory C-reactive protein and creatinine kinase in blood after intake of *Rhodiola rosea*.	Thirty-six volunteers aged 21–24 years participated in the study. Twelve of them had an intake of 340 mg *Rhodiola rosea* extract twice a day, 12 participants were in the placebo group, and 12 participants were in the control group. Period of study—36 days.	Increased levels of C-reactive protein and creatinine kinase.	[79]
Examine the effects and safety of *Rhodiola rosea* extract for 4 weeks of treatment.	There were 101 women and men, aged 30–60 years, who participated in study. All participants had an intake of *Rhodiola rosea* extract 400 mg/day. Period of the study—1 month.	Improved mood, stress symptoms, and quality of life.	[80]
Examine the effects of a single dose of standardized *Rhodiola rosea* extract.	There were 121 men aged 19–21 years participated in the study; 41 participants received 370 mg dry extract *Rhodiola rosea*, 20 participants received 555 mg dry extract of *Rhodiola rosea* before test, 40 of participants were in the placebo group, 20 participants were in the controlled group.	Improvement in the anti-fatigue index.	[81]
Examine the effects of standardized *Rhodiola rosea* extract in patients suffering from depression.	Eighty-nine women and men, aged 18–70 years, participated in the study. Thirty-one participants received 340 mg/day extract of *Rhodiola rosea*, 29 participants received 680 mg/day extract of *Rhodiola rosea*, and 29 participants were in the placebo group. Period of study—42 days.	Improved in overall depression, insomnia, somatization, and emotional instability. No improvements in self-belief.	[82]
Evaluating the changes of *Rhodiola rosea* supplementation on muscle damage and inflammation.	There were 48 men and women, aged 25–60 years, who participated in the study. Twenty-four participants received a 300 mg capsule per day containing *Rhodiola rosea* extract, and 24 participants were in the placebo group. Period of study—38 days.	Increased myoglobin, creatine phosphokinase, aspartate aminotransferase, alanine aminotransferase, and interleukin (IL-6, IL-8, IL-10) without a difference in both groups.	[83]
Examine the effects of *Rhodiola rosea* supplementation on selected redox parameters in athletes.	Twenty-two men aged 20.4 ± 1.2 participated in the study. Eleven of them had an intake of 200 mg/day *Rhodiola rosea* extract, and 11 were in the placebo group.Period of study—4 weeks. Decreased levels of superoxide dismutase. Increased total antioxidant capacity.	Decreased levels of superoxide dismutase. Increased total antioxidant capacity.	[84]
Examine the effects of chronic intake of *Rhodiola rosea* on physical performance and antioxidant capacity during exercise in athletes.	Fourteen men, aged 25 ± 5 years, participated in the study. All of the participants received a placebo; after that, all of them received 170 mg *R. rosea* extract for 1 month.	Decreased free fatty acids levels, blood lactate, and creatinine kinase levels. No change in VO2max.	[85]
The efficacy of *Rhodiola rosea* in generalized anxiety disorder.	Ten men and women, aged 34–55 years, participated in the study. All participants had intake 340 mg *Rhodiola rosea* extract per day for 10 weeks.	Decreased scores in Hamilton Anxiety Rating Scale and Hamilton Depression Rating Scale.	[86]

**Table 6:** *Schisandra chinensis* studies.

Study Objectives	Study Design	Main Results	References
Examine the effects of *Schisandra chinensis* extract on muscle strength and lactate.	Forty-five volunteers aged 61.9 ± 8.4 years participated in the study. Twenty-four participants received 1000 mg/day extract of *Schisandra chinensis* and 21 participants were in the placebo group. Period of study—3 months.	Decreased lactate levels. Increased quadriceps and muscle strength.	[105]
Examine the effects of *Schisandra chinensis* extract for menopausal symptoms.	Thirty-six women aged 40–70 years participated in the study. Eighteen participants received *Schisandra chinensis* extract 748 mg twice a day for 6 weeks; 18 were in the placebo group. Period of study—12 weeks.	Decreased hot flushes, sweating, and heart rate.	[106]
Examine the effects of *Schisandra chinensis* fruit on gut microbiota.	Twenty-eight women participated in the study. Thirteen participants received 6.7 g/day dried *Schisandra chinensis* fruits, and 15 participants were in placebo group. Period of study—12 weeks.	Decreased blood glucose, triglycerides, alanine aminotransferase, aspartate aminotransferase, and fat mass. Increased Bacteroides and Bacteroidetes.	[107]

**Table 7:** *Schisandra chinensis* animal studies.

Study Objectives	Study Design	Main Results	References
Examine the anti-athletics fatigue effects of *Schisandra chinensis*.	For the study, eight mice were used, divided into five groups—the control group, low-dose group—treated with 15 mg/kg *Schisandra chinensis* aqueous extract; the medium-dose group—treated with 30 mg/kg *Schisandra chinensis* aqueous extract; intermediate-high group—treated with 50 mg/kg *Schisandra chinensis* aqueous extract; and high-group—treated with 80 mg/kg *Schisandra chinensis* aqueous extract for 28 days.	Prevented an increase of lactate levels and blood urea nitrogen. Increased blood hemoglobin levels.	[108]
Examine *Schisandra chinensis* effects on pituitary-adrenal and gonadal axis, interleukins, and blood glucose levels.	For the study, 45 rats were used, aged 6 weeks old. There were 15 mice in the control group, 15 in the stress group, and 15 in the group that received 5 g/kg/day *Schisandra chinensis* and exercise. Period of study—11 days.	Decreased blood glucose levels, cortisol, and interleukins (IL-1 and IL-2).	[109]
Examine *Schisandra chinensis* effects on atherosclerosis in rats.	For the study, there were 60 rats used, aged 4 weeks, and 20 mice, aged 6 weeks. They were divided into five groups—normal, model, simvastatin (received 4 mg/kg/day), and low-dose group—which received an extract of *Schisandra chinensis*, 0.35 mg/kg day; medium-dose group—received extract of *Schisandra chinensis* 0.7 mg/kg/day; and high-dose group—received 1.4 mg/kg/day *Schisandra chinensis* extract for 3 weeks. Period of study—12 weeks.	Decreased TG and LDL levels. Increased HDL.	[110]
Examine the effect of *Schisandra chinensis* on mice with hyperlipidemia.	For the study, 48 mice were used. There were 24 mice in the control group and 24 received 100 mg/kg/day *Schisandra chinensis* lignans for 4 weeks.	Decreased TC, TG, LDL. Increased HDL, inhibited the mRNA expression of liver X receptor alfa and the mRNA expression level of hepatic lipogenesis.	[111]
Examine Schisandrine B effects on hepatic glutathione antioxidant system.	For the study, mice were used, with no information for number of mice. They were divided into five groups. Control group and the rest had received Schisandrine B 1 to 4 mmol/kg for 3 days; after that, they were threatened with CCl4 0.1 mL/kg.	Decreased glucose-6-phosphate dehydrogenase, gama-glutamilcysteine synthetase, and Se-glutathione peroxidase. Increased hepatic gluthatione S-transferase and glutathione reductase.	[112]
Examine the preventive effect of Schisandrine B on scopolamine-induced dementia in mice.	For the study, 30 mice were used, aged 12–14 weeks. They were divided into groups—the control group, three groups that had received Schisandrine B, respectively, 10, 25, or 50 mg/kg/daily before treatment with scopolamine, and a group that had received 10 mg/kg/day tacrine. Period of study—7 days.	Prevented scopolamine-induced oxidative stress. Prevented the decrease of acetylcholine levels.	[113]
Evaluated muscle-protective effects of *Schisandra chinensis* extract in mice, after exercise.	For the study, 48 mice were used, aged 10 months. They were divided into six groups with eight mice in each group. The first group received distilled water. The second group received distilled water with exercise control. The third group received 50 mg/kg oxymetholone. The rest of the three groups received, respectively, 125, 250, or 500 mg/kg per day of *Schisandra chinensis* extract and exercises. Period of study—28 days.	Decreased creatine, creatine kinase, and lactate dehydrogenase. Increased myofibre diameter. Inhibited lipid peroxidation, reactive oxygen species.	[114]
Examine the immunostimulatory effect of polysaccharides from *Schisandra chinensis*.	For the study, 50 mice were used, divided into five groups. Three of the groups were administered with 50, 100, or 200 mg/kg/day *Schisandra chinensis* polysaccharides IIa; the other two groups were the control and model groups. Period of study—10 days.	Increased the phagocytic activity of peritoneal macrophages and lymphocyte transformation.	[115]
Examine the anti-diabetic effect of polysaccharides from *Schisandra chinensis*.	For the study, 60 mice were used, divided into six groups: control group, control group with diabetes, alloxan-induced diabetic mice treated with sodium chloride solution, and three groups treated with 162, 324, or 648 mg/kg *Schisandra chinensis* polysaccharides, and a placebo group. Period of study—21 days.	Decreased blood glucose levels. Improved lipid metabolism.	[116]

## References

[1] Wagner H., Nörr H., Winterhoff H. (1994). Plant adaptogens. Phytomedicine.

[2] Panossian A., Wikman G., Wagner H. (1999). Plant adaptogens III. Earlier and more recent aspects and concepts on their mode of action. Phytomedicine.

[3] Oliynyk S., Oh S.-K. (2012). The pharmacology of Actoprotectors: Practical application for improvement of mental and physical performance. Biomol. Ther..

[4] Panossian A.G., Efferth T., Shikov A.N., Pozharitskaya O.N., Kuchta K., Mukherjee P.K., Banerjee S., Heinrich M., Wu W., Guo D. (2020). Evolution of the adaptogenic concept from traditional use to medical systems: Pharmacology of stress and aging related diseases. Med. Res. Rev..

[5] The World Anti-Doping Agency—WADA Executive Committee Approved the List of Prohibited Substances and Methods for 2009. https://www.wada-ama.org/en/media/news/2008-09/wada-executive-committee-approves-2009-prohibited-list-new-delhi-laboratory-0.

[6] The World Anti-Doping Agency—WADA (2018). Prohibited List. https://www.wada-ama.org/sites/default/files/prohibited_list_2018_en.pdf.

[7] Brekhman A.I., Dardymov I.V. (1969). New substances of plant origin which increase nonspecific resistance. Annu. Rev. Pharmacol..

[8] Kelly G.S. (2001). Rhodiola rosea: A possible plant adaptogen. Altern. Med. Rev..

[9] Kamal M., Arif M., Jawaid T. (2017). Adaptogenic medicinal plants utilized for strengthening the power of resistance during chemotherapy—A review. Orient. Pharm. Exp. Med..

[10] Panossian A., Wikman G., Kaur P., Asea A. (2009). Adaptogens exert a stress-protective effect by modulation of expression of molecular chaperones. Phytomedicine.

[11] Pawar V.S., Shivakumar H. (2012). A current status of adaptogens: Natural remedy to stress. Asian Pac. J. Trop. Dis..

[12] Li Z., He X., Liu F., Wang J., Feng J. (2018). A review of polysaccharides from Schisandra chinensis and Schisandra sphenanthera: Properties, functions and applications. Carbohydr. Polym..

[13] Hikino H., Takahashi M., Otake K., Konno C. (1986). Isolation and Hypoglycemic Activity of Eleutherans A, B, C, D, E, F, and G: Glycans of Eleutherococcus senticosus Roots. J. Nat. Prod..

[14] Kokoska L., Janovska D. (2009). Chemistry and pharmacology of Rhaponticum carthamoides: A review. Phytochemistry.

[15] Panossian A. (2017). Understanding adaptogenic activity: Specificity of the pharmacological action of adaptogens and other phytochemicals. Ann. N. Y. Acad. Sci..

[16] Mendes F.R., Carlini E. (2007). Brazilian plants as possible adaptogens: An ethnopharmacological survey of books edited in Brazil. J. Ethnopharmacol..

[17] Ajala T.O. (2017). The effects of adaptogens on the physical and psychological symptoms of chronic stress. DISCOV. Ga. State Honor. Coll. Undergrad. Res. J..

[18] Domene A.M. (2013). Effects of adaptogen supplementation on sport performance. A recent review of published studies. J. Hum. Sport Exerc..

[19] Krasutsky A.G., Cheremisinov V.N. The use of Levzey’s extract to increase the efficiency of the training process in fitness clubs students. Proceedings of the Actual Problems of Biochemistry and Bioenergy of Sport of the XXI Century.

[20] Aslanyan G., Amroyan E., Gabrielyan E., Nylander M., Wikman G., Panossian A. (2010). Double-blind, placebo-controlled, randomised study of single dose effects of ADAPT-232 on cognitive functions. Phytomedicine.

[21] Reay J.L., Scholey A., Kennedy D. (2010). Panax ginseng (G115) improves aspects of working memory performance and subjective ratings of calmness in healthy young adults. Hum. Psychopharmacol. Clin. Exp..

[22] Page M.J., McKenzie J., Bossuyt P.M., Boutron I., Hoffmann T.C., Mulrow C.D., Shamseer L., Tetzlaff J.M., Akl E., Brennan S. (2021). The PRISMA 2020 statement: An updated guideline for reporting systematic reviews. BMJ.

[23] Baeg I.-H., So S.-H. (2013). The world ginseng market and the ginseng (Korea). J. Ginseng Res..

[24] Kiefer D.S., Pantuso T. (2003). Panax ginseng. Am. Fam. Physician.

[25] Patel S., Rauf A. (2016). Adaptogenic herb ginseng (Panax) as medical food: Status quo and future prospects. Biomed. Pharmacother..

[26] Shergis J., Zhang A.L., Zhou W., Xue C.C. (2012). Panax ginseng in randomised controlled trials: A systematic review. Phytother. Res..

[27] Nocerino E., Amato M., Izzo A. (2000). The aphrodisiac and adaptogenic properties of ginseng. Fitoterapia.

[28] Mahady G.B., Gyllenhaal C., Fong H.H., Farnsworth N.R. (2000). Ginsengs: A review of safety and efficacy. Nutr. Clin. Care.

[29] Wilson L. (2007). Review of adaptogenic mechanisms: Eleuthrococcus senticosus, panax ginseng, rhodiola rosea, schisandra chinensis and withania somnifera. Aust. J. Med. Herbal..

[30] Christensen L.P. (2008). Ginsenosides: Chemistry, biosynthesis, analysis, and potential health effects. Adv. Food Nutr. Res..

[31] European Directorate for the Quality of Medicines & Health Care (2019). Ginseng radix. European Pharmacopoeia, Monograph 07/2019:1523.

[32] Kim S.H., Park K.S. (2003). Effects of panax ginseng extract on lipid metabolism in humans. Pharmacol. Res..

[33] Bhattacharjee I., Bandyopadhyay A. (2020). Effects of acute supplementation of panax ginseng on endurance performance in healthy adult males of Kolkata, India. Int. J. Clin. Exp. Physiol..

[34] Etemadifar M., Sayahi F., Abtahi S.-H., Shemshaki H., Dorooshi G.-A., Goodarzi M., Akbari M., Fereidan-Esfahani M. (2013). Ginseng in the treatment of fatigue in multiple sclerosis: A randomized, placebo-controlled, double-blind pilot study. Int. J. Neurosci..

[35] Engels H.-J., Said J.M., Wirth J.C. (1996). Failure of chronic ginseng supplementation to affect work performance and energy metabolism in healthy adult females. Nutr. Res..

[36] Perazzo F.F., Fonseca F.L., Souza G.H.B., Maistro E.L., Rodrigues M., Carvalho J.C. (2010). Double-blind clinical study of a multivitamin and polymineral complex associated with panax ginseng extract (Gerovital®). Open Complement. Med. J..

[37] Ziemba A.W. The effect of ginseng supplementation on psychomotor performance, indices of physical capacity and plasma concentration of some hormones in young well fit men. Proceedings of the Ginseng Society Conference.

[38] Zarabi L., Arazi H., Izadi M. (2018). The effects of panax ginseng supplementation on growth hormone, cortisol and lactate response to high-intensity resistance exercise. Biomed. Hum. Kinet..

[39] Lee S.A., Kang S.G., Lee H.J., Jung K.Y., Kim L. (2010). Effect of Korean red ginseng on sleep: A randomized, placebo-controlled Trial. Sleep Med. Psychophysiol..

[40] Kim H.-G., Cho J.-H., Yoo S.-R., Lee J.-S., Han J.-M., Lee N.-H., Ahn Y.-C., Son C.-G. (2013). Antifatigue Effects of Panax ginseng CA Meyer: A randomised, double-blind, placebo-controlled trial. PLoS ONE.

[41] Ping F.W.C., Keong C.C., Bandyopadhyay A. (2011). Effects of acute supplementation of Panax ginseng on endurance running in a hot & humid environment. Indian J. Med. Res..

[42] Davydov M., Krikorian A. (2000). Eleutherococcus senticosus (Rupr. & Maxim.) maxim. (Araliaceae) as an adaptogen: A closer look. J. Ethnopharmacol..

[43] World Health Organization (2002). WHO Monographs on Selected Medicinal Plants.

[44] Bleakney T.L. (2008). Deconstructing an adaptogen: Eleutherococcus Senticosus. Holist. Nurs. Pract..

[45] Jia A., Zhang Y., Gao H., Zhang Z., Zhang Y., Wang Z., Zhang J., Deng B., Qiu Z., Fu C. (2020). A review of Acanthopanax senticosus (Rupr and Maxim.) harms: From ethnopharmacological use to modern application. J. Ethnopharmacol..

[46] Asano K., Takahashi T., Miyashita M., Matsuzaka A., Muramatsu S., Kuboyama M., Kugo H., Imai J. (1986). Effect of eleutheroccocus senticosus extract on human physical working capacity. Planta Med..

[47] European Directorate for the Quality of Medicines & Health Care (2016). Eleutherococci radix. European Pharmacopoeia, Monograph 01/2008:1419.

[48] Dowling E.A., Redondo D.R., Branch J.D., Jones S., McNabb G., Williams M.H. (1996). Effect of Eleutherococcus senticosus on submaximal and maximal exercise performance. Med. Sci. Sports Exerc..

[49] Kuo J., Chen K.W., Cheng I.S., Tsai P.H., Lu Y.J., Lee N.Y. (2010). The effect of eight weeks of supplementation with Eleutherococcus senticosus on endurance capacity and metabolism in human. Chin. J. Physiol..

[50] Cicero A., DeRosa G., Brillante R., Bernardi R., Nascetti S., Gaddi A. (2004). effects of siberian ginseng (eleutherococcus senticosus maxim.) on elderly quality of life: A randomized clinical trial. Arch. Gerontol. Geriatr..

[51] Szołomicki S., Samochowiec L., Wójcicki J., Droździk M. (2000). The influence of active components of eleutherococcus senticosus on cellular defence and physical fitness in man. Phytother. Res..

[52] Schaffler K., Wolf O., Burkart M. (2013). No Benefit Adding Eleutherococcus senticosus to Stress Management Training in Stress-Related Fatigue/Weakness, Impaired Work or Concentration, A Randomized Controlled Study. Pharmacopsychiatry.

[53] Eschbach L.C., Webster M.J., Boyd J.C., McArthur P.D., Evetovich T.K. (2000). The Effect of Siberian Ginseng (Eleutherococcus Senticosus) on Substrate Utilization and Performance during Prolonged Cycling. Int. J. Sport Nutr. Exerc. Metab..

[54] Krasutsky A.G., Cheremisinov V.N. Research of the influence of adaptogens on increasing the efficacy of the training process in fitness clubs. Proceedings of the Current Problems of Biochemistry and Bioenergy Sport of the XXI Centyry.

[55] Jacquet A., Grolleau A., Jove J., Lassalle R., Moore N. (2015). Burnout: Evaluation of the efficacy and tolerability of TARGET 1® for professional fatigue syndrome (burnout). J. Int. Med. Res..

[56] Buděšínský M., Vokáč K., Harmatha J., Cvačka J. (2008). Additional minor ecdysteroid components of Leuzea carthamoides. Steroids.

[57] Timofeev N.P., Martirosyan D.M. (2006). Leuzea Carthamoides DC: Application prospects as pharmpreparations and biologically active components. Functional Foods for Chronic Diseases.

[58] Bathori M., Toth N., Hunyadi A., Marki A., Zador E. (2008). Phytoecdysteroids and anabolic-androgenic steroids—Structure and effects on humans. Curr. Med. Chem..

[59] Isenmann E., Ambrosio G., Joseph J.F., Mazzarino M., de la Torre X., Zimmer P., Kazlauskas R., Goebel C., Botrè F., Diel P. (2019). Ecdysteroids as non-conventional anabolic agent: Performance enhancement by ecdysterone supplementation in humans. Arch. Toxicol..

[60] The World Anti-Doping Agency—WADA The 2020 Monitoring Program. https://www.wada-ama.org/sites/default/files/resources/files/wada_2020_english_monitoring_program_pdf.

[61] Vanyuk A.I. (2012). Evaluation of the effectivnness of rehabilitation measures among female volleyball players 18-22 years old in the competitive period of the annual training cycle. Slobozhanskiy Sci. Sports Visnik..

[62] Timofeev N.P., Koksharov A.V. (2016). Study of Leuzea from leaves: Results of 15 years of trials in athletics. New Unconv. Plants Prospect. Use.

[63] Wilborn C.D., Taylor L.W., Campbell B.I., Kerksick C., Rasmussen C.J., Greenwood M., Kreider R.B. (2006). Effects of Methoxyisoflavone, ecdysterone, and sulfo-polysaccharide supplementation on training adaptations in resistance-trained males. J. Int. Soc. Sports Nutr..

[64] Ryan E.D., Gerstner G.R., Mota J.A., Trexler E.T., Giuliani H.K., Blue M.N.M., Hirsch K.R., Smith-Ryan A.E. (2020). The acute effects of a multi-ingredient herbal supplement on performance fatigability: A double-blind, randomized, and placebo-controlled trial. J. Diet. Suppl..

[65] Selepcova L., Sommer A., Vargova M. (2013). Effect of feeding on a diet containing varying amounts of rhaponticum car-thamoides hay meal on selected morphological parameters in rats. Eur. J. Entornol..

[66] Plotnikov M.B., Aliev O.I., Vasil’Ev A.S., Andreeva V.Y., Krasnov E.A., Kalinkina G.I. (2008). Effect of Rhaponticum carthamoides extract on structural and metabolic parameters of erythrocytes in rats with cerebral ischemia. Bull. Exp. Biol. Med..

[67] Wu J., Gao L., Shang L., Wang G., Wei N., Chu T., Chen S., Zhang Y., Huang J., Wang J. (2017). Ecdysterones from Rhaponticum carthamoides (Willd.) Iljin reduce hippocampal excitotoxic cell loss and upregulate mTOR signaling in rats. Fitoter.

[68] Seidlova-Wuttke D., Ehrhardt C., Wuttke W. (2010). Metabolic effects of 20-OH-Ecdysone in ovariectomized rats. J. Steroid Biochem. Mol. Biol..

[69] Koudela K., Tenora J., Bajer J., Mathova A., Slama K. (1995). Stimulation of growth and development in Japanase quails after oral administration of ecdysteroid-containing diet. Eur. J. Entomol..

[70] Sláma K., Koudela K., Tenora J., Maťhová A. (1996). Insect hormones in vertebrates: Anabolic effects of 20-hydroxyecdysone in Japanese quail. Experientia.

[71] Xia X., Zhang Q., Liu R., Wang Z., Tang N., Liu F., Huang G., Jiang X., Gui G., Wang L. (2014). Effects of 20-hydroxyecdysone on improving memory deficits in streptozotocin-induced type 1 diabetes mellitus in rat. Eur. J. Pharmacol..

[72] Roumanille R., Vernus B., Brioche T., Descossy V., Van Ba C.T., Campredon S., Philippe A.G., Delobel P., Bertrand-Gaday C., Chopard A. (2020). Acute and chronic effects of Rhaponticum carthamoides and Rhodiola rosea extracts supplementation coupled to resistance exercise on muscle protein synthesis and mechanical power in rats. J. Int. Soc. Sports Nutr..

[73] Brown R.P., Gerbarg P.L., Ramazanov Z. (2002). Rhodiola rosea: A phytomedicinal overview. Herbal. Gram..

[74] Pu W.-L., Zhang M.-Y., Bai R.-Y., Sun L.-K., Li W.-H., Yu Y.-L., Zhang Y., Song L., Wang Z.-X., Peng Y.-F. (2020). Anti-inflammatory effects of Rhodiola rosea L.: A review. Biomed. Pharmacother..

[75] Khanum F., Bawa A.S., Singh B. (2005). Rhodiola rosea: A versatile adaptogen. Compr. Rev. Food Sci. Food Saf..

[76] Panossian A., Seo E.-J., Efferth T. (2018). Novel molecular mechanisms for the adaptogenic effects of herbal extracts on isolated brain cells using systems biology. Phytomedicine.

[77] Ballmann C.G., Maze S.B., Wells A.C., Marshall M.R., Rogers R.R. (2019). Effects of short-term Rhodiola Rosea (golden root extract) supplementation on anaerobic exercise performance. J. Sports Sci..

[78] Jówko E., Sadowski J., Długołęcka B., Gierczuk D., Opaszowski B., Cieśliński I. (2018). Effects of Rhodiola rosea supplementation on mental performance, physical capacity, and oxidative stress biomarkers in healthy men. J. Sport Health Sci..

[79] Abidov M., Grachev S., Seifulla R.D., Ziegenfuss T.N. (2004). Extract of Rhodiola rosea radix reduces the level of c-reactive protein and creatinine kinase in the blood. Bull. Exp. Biol. Med..

[80] Edwards D., Heufelder A., Zimmermann A. (2012). Therapeutic effects and safety of Rhodiola rosea extract WS® 1375 in subjects with life-stress symptoms—Results of an open-label study. Phytother. Res..

[81] Shevtsov V., Zholus B., Shervarly V., Vol’Skij V., Korovin Y., Khristich M., Roslyakova N., Wikman G. (2003). A randomized trial of two different doses of a SHR-5 Rhodiola rosea extract versus placebo and control of capacity for mental work. Phytomedicine.

[82] Darbinyan V., Aslanyan G., Amroyan E., Gabrielyan E., Malmström C., Panossian A. (2007). Clinical trial of Rhodiola rosea L. extract SHR-5 in the treatment of mild to moderate depression. Nord. J. Psychiatry.

[83] Shanely R.A., Nieman D.C., Zwetsloot K.A., Knab A.M., Imagita H., Luo B., Davis B., Zubeldia J.M. (2013). Evaluation of Rhodiola rosea supplementation on skeletal muscle damage and inflammation in runners following a competitive marathon. Brain Behav. Immun..

[84] Stejnborn A.S., Pilaczyńska-Szcześniak S., Basta P., Deskur-Śmielecka E. (2009). The influence of supplementation with Rhodiola rosea L. Extract on selected redox parameters in professional rowers. Int. J. Sport Nutr. Exerc. Metab..

[85] Parisi A., Tranchita E., Duranti G., Ciminelli E., Quaranta F., Ceci R., Sabatini S. (2010). Effects of chronic Rhodiola Rosea sup-plementation on sport performance and antioxidant capacity in trained male: Preliminary results. J. Sports Med. Phys. Fit..

[86] Bystritsky A., Kerwin L., Feusner J.D. (2008). A pilot study of Rhodiola rosea (Rhodax®) for generalized anxiety disorder (GAD). J. Altern. Complement. Med..

[87] Hancke J., Burgos R., Ahumada F. (1999). Schisandra chinensis (Turcz.) Baill. Fitoterapia.

[88] Lu Y., Chen D.-F. (2009). Analysis of Schisandra chinensis and Schisandra sphenanthera. J. Chromatogr. A.

[89] Panossian A., Wikman G. (2008). Pharmacology of Schisandra chinensis bail.: An overview of Russian research and uses in medicine. J. Ethnopharmacol..

[90] Slanina J., Táborská E., Lojková L. (1997). Lignans in the seeds and fruits of Schisandra chinensis cultured in Europe. Planta Med..

[91] European Directorate for the Quality of Medicines & Health Care (2016). Schisandrae chinensis fructus. European Pharmacopoeia, Monograph 07/2016:2428.

[92] Szopa A., Barnaś M., Ekiert H. (2019). Phytochemical studies and biological activity of three Chinese Schisandra species (Schisandra sphenanthera, Schisandra henryi and Schisandra rubriflora): Current findings and future applications. Phytochem. Rev..

[93] Kochetkov N., Khorlin A., Chizhov O., Sheichenko V. (1961). Schizandrin—Lignan of unusual structure. Tetrahedron Lett..

[94] Chen D.-F., Zhang S.-X., Kozuka M., Sun Q.-Z., Feng J., Wang Q., Mukainaka T., Nobukuni Y., Tokuda H., Nishino H. (2002). Interiotherins C and D, two new lignans from Kadsurainteriorand antitumor-promoting effects of related neolignans on Epstein−Barr Virus Activation. J. Nat. Prod..

[95] Yoo H.H., Lee M., Lee M.W., Lim S.Y., Shin J., Kim D.-H. (2007). Effects of Schisandra lignans on P-Glycoprotein-mediated drug efflux in human intestinal Caco-2 Cells. Planta Med..

[96] Fong W.-F., Wan C.-K., Zhu G.-Y., Chattopadhyay A., Dey S., Zhao Z., Shen X.-L. (2007). Schisandrol A from Schisandra chinensis reverses P-Glycoprotein-mediated multidrug resistance by affecting Pgp-substrate complexes. Planta Med..

[97] Chen M., Kilgore N., Lee K.-H., Chen D.-F. (2006). Rubrisandrins A and B, lignans and related anti-HIV compounds from Schisandra rubriflora. J. Nat. Prod..

[98] Chen D.-F., Zhang S.-X., Xie L., Xie J.-X., Chen K., Kashiwada Y., Zhou B.-N., Wang P., Cosentino L., Lee K.-H. (1997). Anti-aids agents—XXVI. Structure-activity correlations of Gomisin-G-related anti-HIV lignans from Kadsura interior and of related synthetic analogues. Bioorganic Med. Chem..

[99] Liu C., Zhang S., Zhang J., Liang Q., Li D. (2012). Chemical composition and antioxidant activity of essential oil from berries of Schisandra chinensis(Turcz.) Baill. Nat. Prod. Res..

[100] Chen X., Zhang Y., Zu Y., Yang L. (2012). Chemical composition and antioxidant activity of the essential oil of Schisandra chinensisfruits. Nat. Prod. Res..

[101] Xu M., Yan T., Gong G., Wu B., He B., Du Y., Xiao F., Jia Y. (2020). Purification, structural characterization, and cognitive improvement activity of a polysaccharides from Schisandra chinensis. Int. J. Biol. Macromol..

[102] Liu Y., Guo J.-T., Wang Z.-B., Li Z.-Y., Zheng G.-X., Xia Y.-G., Yang B.-Y., Kuang H.-X. (2019). Aromatic monoterpenoid glycosides from rattan stems of Schisandra chinensis and their neuroprotective activities. Fitoterapia.

[103] Mocan A., Crișan G., Vlase L., Crișan O., Vodnar D.C., Raita O., Gheldiu A.-M., Toiu A., Oprean R., Tilea I. (2014). Comparative studies on polyphenolic composition, antioxidant and antimicrobial activities of schisandra chinensis leaves and fruits. Molecules.

[104] Yang B.-Y., Guo J.-T., Li Z.-Y., Wang C.-F., Wang Z.-B., Wang Q.-H., Kuang H.-X. (2016). New Thymoquinol Glycosides and Neuroprotective Dibenzocyclooctane Lignans from the Rattan Stems ofSchisandra chinensis. Chem. Biodivers..

[105] Park J., Han S., Park H. (2020). Effect of Schisandra chinensis extract supplementation on quadriceps muscle strength and fatigue in adult women: A randomized, double-blind, placebo-controlled trial. Int. J. Environ. Res. Public Health.

[106] Park J.Y., Kim K.H. (2016). A randomized, double-blind, placebo-controlled trial of Schisandra chinensis for menopausal symptoms. Climacteric.

[107] Song M.-Y., Wang J., Eom T., Kim H. (2015). Schisandra chinensis fruit modulates the gut microbiota composition in association with metabolic markers in obese women: A randomized, double-blind placebo-controlled study. Nutr. Res..

[108] Cao S., Shang H., Wu W., Du J., Putheti R. (2009). Evaluation of anti-athletic fatigue activity of Schizandra chinensis aqueous extracts in mice. Afr. J. Pharm. Pharmacol..

[109] Li J., Wang J., Shao J.-Q., Du H., Wang Y.-T., Peng L. (2014). Effect of Schisandra chinensis on interleukins, glucose metabolism, and pituitary-adrenal and gonadal axis in rats under strenuous swimming exercise. Chin. J. Integr. Med..

[110] Chen X., Cao J., Sun Y., Dai Y., Zhu J., Zhang X., Zhao X., Wang L., Zhao T., Li Y. (2018). Ethanol extract of Schisandrae chinensis fructus ameliorates the extent of experimentally induced atherosclerosis in rats by increasing antioxidant capacity and improving endothelial dysfunction. Pharm. Biol..

[111] Sun J.-H., Liu X., Cong L.-X., Li H., Zhang C.-Y., Chen J.-G., Wang C.-M. (2017). Metabolomics study of the therapeutic mechanism of Schisandra chinensis lignans in diet-induced hyperlipidemia mice. Lipids Health Dis..

[112] Ip S.-P., Poon M., Wu S., Che C., Ng K., Kong Y., Ko K. (1995). Effect of Schisandrin B on hepatic glutathione antioxidant system in mice: Protection against carbon tetrachloride toxicity. Planta Med..

[113] Giridharan V.V., Thandavarayan R.A., Sato S., Ko K.M., Konishi T. (2011). Prevention of scopolamine-induced memory deficits by schisandrin B, an antioxidant lignan from Schisandra chinensis in mice. Free. Radic. Res..

[114] Kim K.-Y., Ku S.-K., Lee K.-W., Song C.-H., An W.G. (2018). Muscle-protective effects of Schisandrae fructus extracts in old mice after chronic forced exercise. J. Ethnopharmacol..

[115] Chen Y., Tang J., Wang X., Sun F., Liang S. (2011). An immunostimulatory polysaccharide (SCP-IIa) from the fruit of Schisandra chinensis (Turcz.) Baill. Int. J. Biol. Macromol..

[116] Zhao T., Mao G.-H., Zhang M., Li F., Zou Y., Zhou Y., Zheng W., Zheng D.-H., Yang L.-Q., Wu X.-Y. (2012). Anti-diabetic effects of polysaccharides from ethanol-insoluble residue of Schisandra chinensis (Turcz.) baill on alloxan-induced diabetic mice. Chem. Res. Chin. Univ..

